# Linseed, Walnut, and Algal Oil Emulsion Gels as Fat Replacers in Chicken Frankfurters: Effects on Composition, Lipid Profile and Sensory Quality

**DOI:** 10.3390/foods14152677

**Published:** 2025-07-30

**Authors:** Tamara Stamenić, Vanja Todorović, Maja Petričević, Tanja Keškić, Bogdan Cekić, Nenad Stojiljković, Nikola Stanišić

**Affiliations:** 1 Institute for Animal Husbandry, Belgrade—Zemun, Autoput za Zagreb 16, 11080 Belgrade, Serbia; tstamenic169@gmail.com (T.S.); majanovakovic@live.com (M.P.); tanjakeskic@gmail.com (T.K.); bogdancekic@gmail.com (B.C.); nstojiljkovic.izs@afrodita.rcub.bg.ac.rs (N.S.); 2 Department of Bromatology, Faculty of Pharmacy, University of Belgrade, Vojvode Stepe 450, 11221 Belgrade, Serbia; vanja.todorovic@pharmacy.bg.ac.rs

**Keywords:** chicken frankfurters, fat substitution, vegetable oils, lipid oxidation, fatty acid profile, sensory evaluation

## Abstract

The replacement of animal fat with unsaturated lipid sources in processed meats enhances nutritional value but introduces challenges regarding oxidative stability and sensory acceptability. In this study, the effects of replacing pork back fat with pre-emulsified walnut, linseed, or algal oils on the proximate composition, fatty acid profile, nutritional indices, lipid oxidation, and sensory properties of chicken frankfurters were investigated. Four formulations were prepared: a control group (25% pork fat) and three groups that were completely reformulated using oil emulsions (ratio inulin/water/oil 1:2:1). The fat substitute significantly reduced total fat, SFA, cholesterol (up to 30%), and calorie density, while Ʃn-3 fatty acids were enriched (*p* < 0.05). The linseed oil samples had the highest levels of α-linolenic acid (47.53%), while the algal oil had the highest levels of eicosapentaenoic acid (10.98%) and docosahexaenoic acid (64.73%) and the most favourable Ʃn-6/Ʃn-3 ratio (*p* < 0.05). All reformulated groups showed significantly improved atherogenic and thrombogenic indices and increased hypocholesterolaemic/hypercholesterolaemic ratios, which reached 17.43 in the algal oil samples (*p* < 0.05). Lipid oxidation was increased in the linseed and algal oil treatments, with the walnut oil group showing moderate TBARS levels and minimal accumulation of secondary oxidation products. Principal component analysis revealed that walnut oil offered the most balanced compromise between nutritional improvement, oxidative stability and sensory acceptability. These findings support a healthier reformulation of meat products by identifying oil-based fat substitutes that improve nutritional value without compromising sensory quality, which is beneficial for both research and industry.

## 1. Introduction

Frankfurters are popular comminuted meat products that are consumed all over the world. Traditional recipes usually use pork and beef, but some variants also contain poultry instead [1]. These types of sausages typically contain up to 30% fat, which contributes significantly to product quality by forming stable emulsions, reducing cooking losses, improving water-holding capacity, enhancing rheological and textural properties, and providing essential sensory characteristics such as juiciness and flavour [1,2]. However, a high consumption of saturated fats and cholesterol from animal sources is associated with an increased incidence of obesity, elevated blood pressure and cardiovascular and coronary heart disease [1,3,4].

Given these health concerns, consumer preferences have shifted towards meat products with reduced or improved fat content while maintaining the desired sensory properties [5,6]. This has led the meat industry to develop alternatives to meat products where animal fat is replaced by healthier lipid sources such as vegetable oils [1,7,8,9]. As they contain no cholesterol and have a higher proportion of unsaturated fatty acids, vegetable oils are associated with better health outcomes [10,11,12].

Numerous studies have investigated the effects of adding vegetable oils such as olive, sunflower, rapeseed, chia, linseed and walnut oil to sausages and frankfurters and have often achieved improved lipid profiles without significant impairment of sensory quality [1,3,13,14,15,16,17]. Walnut, chia and linseed oils have been highlighted for their ability to reduce SFA content and enrich n-3 fatty acids [1,17,18]. Replacing animal fats with marine and vegetable oils can significantly improve the fatty acid profile by reducing the SFA content and optimising the n-6/n-3 ratio, which is crucial for reducing the risk of metabolic disorders, obesity and coronary heart disease [19,20,21]. Recent dietary guidelines recommend reducing the intake of SFA to less than 10% of daily calories and replacing them with monounsaturated and polyunsaturated fatty acids [22]. Oleic-rich oil-based emulsions and oleogels have shown promise in achieving these nutritional goals while maintaining acceptable texture and sensory profiles [23,24,25]. Algal oil has proven to be a promising fat substitute due to its high content of long-chain n-3 fatty acids (EPA, DHA). Its use in meat products is already being explored: in beef burgers, a “high omega-3 content” was achieved by completely replacing pork cheek fat with algal oil emulsions, significantly improving the ratio of PUFA to SFA and of n-6 to n-3, while the technological and sensory parameters remained acceptable [26]. Similarly, algal oil-based hydrogels in pork burgers have produced fat-reduced products with lower SFA as well as improved moisture, protein and ash content and favourable fatty acid indices, with sensory properties similar to controls [27].

However, the direct incorporation of oils into meat products can pose technological challenges in terms of taste, colour and oxidative stability [11,12]. For this reason, structured lipid systems such as emulsion gels and oleogels have proven to be viable strategies to incorporate vegetable oils into meat matrices while maintaining product quality [18,20,23,28,29]. These structured systems provide mechanical stability and can effectively mimic the textural properties of animal fat [23,24,30,31]. Various gelling agents, including inulin, alginate, carrageenan, konjac and chia mucilage, have been used to stabilise these emulsions [32,33,34,35].

Previous studies have focused on the partial or total replacement of pork back fat in frankfurters and sausages with oils such as rapeseed, olive, sunflower and fish oil [14,18,36]. However, there are few studies focusing on poultry frankfurters, especially those using linseed, walnut, and seaweed oils in textured emulsions. To address these gaps, this study reformulated chicken frankfurters, chosen for their lean profile, healthier consumer perception and growing market demand, by completely replacing the pork backfat with structured emulsions of linseed, walnut and algal oil in a 1:2:1 ratio. Linseed, walnut and algal oils were selected for their high n-3 PUFA content and their potential to improve the nutritional profile of meat products. Linseed and walnut oils improve health indices with minimal impact on texture or flavour, while algal oil provides EPA and DHA and has shown promise to improve fatty acid profiles and sensory acceptability when structured in emulsions. We systematically analysed the composition, cholesterol content, fatty acid profile, and lipid oxidation stability (TBA and AnV) during storage and sensory properties. This integrated approach aimed to develop nutritionally improved frankfurters with a lower cardiovascular risk potential while maintaining consumer acceptance.

## 2. Materials and Methods

### 2.1. Materials and Chemicals

Collagen casing (22 mm diameter) was purchased from Koteks Viscofan (Novi Sad, Serbia). Additives and spices were supplied by Fenc Company (Novi Sad, Serbia). Linseed and walnut oil were kindly donated by Linum doo (Čonoplja, Serbia). Algal oil (Omegatex^®^ALGAE1060TG) was kindly provided by Solutex (Madrid, Spain). Inulin fibres (Fibruline™) were purchased from Cosucra (Warcoing, Belgium). All reagents and chemicals used to analyse the proximate composition, fatty acid profile, oxidation indicators and cholesterol content of frankfurters were of analytical grade.

### 2.2. Frankfurters Preparation and Experimental Set-Up

Chicken frankfurters were produced from skinless chicken breast meat from 500 Cobb hybrid chickens (slaughtered at 42 days of age) and pork back fat from the experimental meat processing plant of the Institute for Animal Husbandry (Belgrade, Serbia). The control formulation (C) consisted of chicken breast meat, pork back fat and water (ice) in a ratio of 50:25:25. In the experimental groups, the pork back fat was completely replaced by structured emulsions based on linseed oil (L), walnut oil (W) or algae oil (A) in a ratio of 1:2:1 (inulin/water/oil). This ratio was selected based on previous studies showing that inulin-based emulsion gels at similar ratios provide desirable stability, water retention, and texture properties when used as fat replacers in processed meat products [30]. The emulsion gels were prepared by first homogenising inulin powder and cold water (4 °C) for 3 min using a high-speed homogeniser (Ultra-Turrax T25, IKA, Staufen im Breisgau, Germany) at 6000 rpm (low speed). The respective oil was then gradually added while increasing the speed to 10,000 rpm (high speed), and mixing continued for an additional 3 min until a stable emulsion was formed.

Each treatment group (C, L, W, A) was prepared in three independent biological replicates with freshly prepared raw materials. All batches were processed on the same day under identical conditions. To avoid systematic bias due to processing order or timing, the order of batch preparation was randomised prior to production using a simple randomisation procedure. Each batch (~5 kg) was prepared by mixing chicken meat, water/ice and the fat source (either pork back fat or oil-based emulsion gel) with salt (1.8%), nitrite curing salt (0.015%), sodium tripolyphosphate (0.4%), sodium erythorbate (0.04%), soy protein isolate (2.0%), sucrose (0.5%), coriander (0.2%), black pepper (0.15%) and garlic powder (0.10%). Soy protein isolate was added in all batches at a constant level of 2.0%, regardless of the fat source or the moisture content of the emulsion. The batter was homogenised using a bowl cutter (Seydelmann K20, Stuttgart, Germany) at two speeds, initial mixing at low speed for 2 min and emulsification at high speed for 5 min, keeping the temperature below 12 °C throughout the process.

The emulsified meat batter was filled into smoke-permeable collagen casings (diameter 22 mm) and cooked in a smoking chamber (Fessmann, Winnenden, Germany) with the following thermal profile: drying at 50 °C for 10 min, smoking at 60 °C for 30 min and heating at 85 °C until the core temperature reached 72 °C. The cooked sausages were cooled under a cold water shower and stored at 4 °C for 24 h. The frankfurters were then placed in polyethylene bags (with low oxygen transmission rate (OTR) of approximately 20 cm^3^/m^2^·day at 23 °C), vacuum packed and kept at 4 ± 2 °C during storage. After 10 days of refrigerated storage, chemical analyses (proximate composition, fatty acid profile, oxidation indicators, and cholesterol content) and sensory evaluation were performed. The evaluation of lipid oxidation (TBARS and AnV values) was determined after one day of refrigerated storage (day 0) and after 15, 30 and 60 days of refrigerated storage under vacuum. All analyses were performed in triplicate for each recipe and nine sausages were randomly selected and analysed in each group.

### 2.3. Proximate Composition and Energy Value

The moisture content was determined by drying the samples at 103 ± 2 °C according to ISO 1442:2023 [37]. The protein content was measured using the Kjeldahl method, whereby the nitrogen content was multiplied by a factor of 6.25 [38]. The fat content was analysed using the Soxhlet extraction method [39]. The ash content was determined by mineralisation of the samples at 550 ± 25 °C [40].

The energy value was calculated on the basis of the following calorie contents: 9 kcal/g for fat and 4 kcal/g for protein and carbohydrates [41].

### 2.4. Fatty Acid Profile and Nutritional Indices

The fatty acids were methylated by direct transesterification and converted to their methyl esters via direct transesterification following the method described by O’Fallon et al. [42]. The resulting organic phase containing the fatty acid methyl esters (FAMEs) was then analysed by gas chromatography (GC). FAMEs were separated using a Shimadzu GC-2014 system (Kyoto, Japan) equipped with a split/splitless injector, a flame ionisation detector (FID), and a fused silica cyanopropyl HP-88 capillary column (60 m × 0.25 mm i.d., 0.20 µm film thickness). The injector and detector temperatures were set at 260 °C and 280 °C, respectively. Helium served as the carrier gas at a flow rate of 1.0 mL/min, with a split ratio of 1:10. A 1 µL sample volume was injected. The oven temperature program began at 50 °C (held for 2 min), increased to 190 °C at 20 °C/min, then to 200 °C at 10 °C/min (held for 10 min), and finally to 250 °C at 15 °C/min (held for 2 min). The GC-FID method was validated prior to analysis: the limit of detection (LOD) ranged from 0.01% to 0.05% of total FAs, the limit of quantification (LOQ) ranged from 0.03% to 0.1%, and the repeatability (expressed as RSD%) was below 5% for all major fatty acids. The chromatographic peaks in the samples were identified by comparing the relative retention times of the FAME peaks with the peaks in a Supelco 37 Component FAMEs Mix Standard (Sigma-Aldrich, Hamburg, Germany). The concentration of each fatty acid was expressed as a percentage of total FA.

The Atherogenic Index (AI) and Thrombogenic Index (TI) were calculated according to Chen and Liu [43], as presented in Equations (1) and (2):

(1) AI = (C12:0 + 4 × C14:0 + C16:0)/(MUFA + PUFA)

(2) TI = (C14:0 + C16:0 + C18:0)/((0.5 × MUFA) + (0.5 × ∑n6) + (3 × ∑n3)+ (∑n3/∑n6))

The hypocholesterolemic/hypercholesterolemic ratio (h/H) was calculated as described by Fernández et al. [44] and presented in Equation (3):

(3) h/H = (∑C18:1 + PUFA)/(C14:0 + C16:0)

### 2.5. Lipid Oxidation Assessment

Lipid oxidation was evaluated using thiobarbituric acid reactive substances (TBARS) and anisidine value (AnV) measurements. The TBARS assay was conducted following the method of Beuge and Aust [45] and the results were expressed as milligrams malondialdehyde (MDA) per 1000 g sample, as presented in Equation (4):

(4) TBARS = ε532 × 2.77,

where TBARS is the thiobarbituric acid reactive substance (mg MDA per 1000 g sample); ε532—absorbance value at 532 nm; 2.77—coefficient. Prior to analyses, a standard curve (0–5 mg MDA L^−1^) was prepared using 1,1,3,3-tetramethoxypropane as the MDA precursor.

The anisidine values (AnV), expressed as the absorbance value, were measured according to ISO 6885 [46] and calculated using the following Formula (5):

(5) AnV = 25 × ((1.2 × ε1 − ε2) − ε0)/m,

where AnV is the anisidine value; ε0 is the absorbance of the sample solution without addition of the p-anisidine reagent; ε1 is the absorbance of the sample solution with addition of the p-anisidine reagent; ε2 is the absorbance of the blank sample; and m is the measured mass (g) of the test sample. AnV was measured spectrophotometrically at 350 nm using a molar absorptivity of 25.0 L g^−1^ cm^−1^ for p-anisidine.

### 2.6. Cholesterol Determination

The cholesterol content was determined by high-performance liquid chromatography with photodiode array detection (HPLC-PDA). The sample was prepared according to the AOAC 994.10 method [47], which was modified in the final step and adapted to the HPLC determination. The chromatographic system was the Alliance 2695 Separation Module with the Photodiode Array Detector 2996, both from Waters, (Milford, MA, USA). The mobile phase consisted of methanol, HPLC-grade (HoneyWell, Riedel-de Haën, Germany), and ultrapure water (ASTM, Type I). The cholesterol content was determined in gradient separation mode on the Kinetex C18 150 × 4.6 mm reversed-phase column from Phenomenex (Torrance, CA, USA). Saponification of the lipids was performed by heating 1 g of homogenised sample with 10 mL ethanolic KOH (1 mol/L) at 80 °C for 30 min, followed by extraction with hexane. No external standard such as 5-α-cholestane was used; cholesterol quantification was based on comparison with a pure cholesterol calibration curve. Cholesterol values were expressed on a wet weight basis and expressed as mg per 100 g of product.

### 2.7. Sensory Evaluation

The sensory evaluation was based on a descriptive analysis by a trained 10-member panel consisting of researchers from the Department of Biomedicine, Technology and Food Safety at the Institute for Animal Husbandry in Belgrade, Serbia. The panellists took part in two training sessions to familiarise themselves with the scale and the definitions of the sensory attributes and to calibrate the evaluation using reference samples. Frankfurters were cooked in a water bath at 75 °C for 30 min, cooled to 22 ± 2 °C (to reflect typical room temperature conditions), cut into quarters (5 × 5 × 3 cm) and served in random order. Samples were coded with three-digit random numbers and evaluated under fluorescent lighting. Panellists rated appearance, colour, aroma, flavour, juiciness, texture, and overall acceptability using a structured 9-point scale (1 = extremely undesirable; 9 = extremely desirable). Water was served between samples to cleanse the palate. Mean values and standard deviations were calculated for each attribute. Inter-individual variability was assessed using analysis of variance, but advanced agreement (e.g., kappa) was not applied as the used scale was continuous and the panel was trained and calibrated prior to assessment.

### 2.8. Statistical Analysis

All statistical analyses were performed using SPSS software, version 22.0 (IBM Corp., Armonk, NY, USA). The data were tested for normality and homogeneity of variances prior to analysis. A one-way analysis of variance (ANOVA) was used to assess the effects of fat source substitution on proximate composition, lipid profile and sensory evaluation. To evaluate the effects of fat source substitution and storage time on fat oxidation parameters, storage time (0, 15, 30, and 60 days) was included as a within-subject (repeated measures) factor in the analysis. If significant differences were found (*p* < 0.05), Tukey’s HSD test was used for post hoc comparisons. Principal component analysis (PCA) was performed to examine the multivariate relationships between proximate composition, fatty acid profiles, cholesterol content, lipid oxidation parameters and sensory characteristics. The suitability of the data for PCA was confirmed by a Kaiser–Meyer–Olkin measure of sampling adequacy of 0.78 and a significant Bartlett’s test of sphericity (*p* < 0.001), indicating an adequate correlation between the variables. A PCA biplot was created using SPSS and graphically refined in Microsoft Excel to visualise clustering patterns. All analyses were based on three independent biological replicates (n = 3) per treatment group. Results are presented as mean values ± standard deviation, and error bars in the figure represent standard deviation.

## 3. Results and Discussion

### 3.1. Proximate Composition and Energy Value

Analysis of proximate composition showed significant changes when pork backfat was replaced with structured emulsions based on linseed, walnut and algal oils (Table 1). Moisture content increased significantly in the reformulated sausages compared to the control (*p* < 0.05), which is consistent with previous studies in which dietary fibre or vegetable oil emulsions increased water retention and emulsion stability [1]. Similar increases in moisture were found when rice bran fibre [48,49], makgeolli lees fibre [36,50] and sunflower seed oil [36] were used. In this study, the increased moisture content is due to the water contained in the inulin-based emulsion gels, which probably improves the water holding capacity, as also observed by Vural et al. [12] and Choi et al. [48]. Inulin is known to retain approximately 3–4 g of water per gram of fibre [30,51], which contributes significantly to the increased moisture content and improved water-holding capacity observed in the emulsion gels. Consequently, protein content was significantly lower in the reformulated samples (*p* < 0.05), which is consistent with the results of Choi et al. [49] showing that increased moisture from fibre or oil emulsions can reduce protein concentration on a relative basis.

Fat content was significantly lower in all reformulated frankfurters (*p* < 0.05), confirming the success of the fat replacement strategy (Table 1). This is consistent with previous reports in which vegetable oil emulsions effectively reduced fat content in sausages and frankfurters [1,12,49]. The fat reduction in our study was more than 30% and was comparable to the reductions achieved when all animal fat was replaced by emulsion gels [23], thus fulfilling the requirement for “reduced fat” according to the European Commission guidelines (2024) [52].

The carbohydrate content increased significantly in the reformulated frankfurters (*p* < 0.05), mainly due to the inulin in the emulsion gels (Table 1). However, the ash content did not differ significantly between treatments (*p* > 0.05), in contrast to the results of Choi et al. [1,48], who found a higher ash content when vegetable oils and fibres were added, probably due to their mineral content.

The energy content of the reformulated frankfurters was significantly lower than that of the control, which is consistent with previous studies showing that vegetable oils and fibre reduce the caloric density of meat products [1,53,54]. The energy reduction in our samples was over 50%, exceeding the typical 20–30% reductions reported in similar formulations [1,23].

Overall, the results confirm that structured emulsions based on linseed, walnut, and algal oils are effective in reducing fat and energy content while increasing moisture and carbohydrate content. These results are in line with the consumer trend towards fat and calorie-reduced meat products and support the potential of these emulsions as effective fat replacers in sausages.

### 3.2. Fatty Acid Profile, Nutritional Indices and Cholesterol Content

The fatty acid composition of frankfurters was significantly altered by replacing pork backfat with structured emulsions based on linseed, walnut, and algal oils (Table 2). Saturated fatty acids (SFA) were highest in the control group (42.02%) and were significantly reduced in all reformulated samples: linseed (13.67%), walnut (13.24%), and algal oil (7.76%) (*p* < 0.05). This considerable reduction is consistent with previous studies in which the replacement of animal fat with vegetable oils or emulsion gels lowered the SFA content in meat products [1,12,23,49]. In particular, palmitic acid (C16:0) and stearic acid (C18:0), the most abundant SFAs in pork backfat and the main contributors to atherogenic potential, were significantly reduced, especially in the algal oil samples [26]. The superior SFA reduction in the algal oil group may be attributed to the unique fatty acid composition of microalgae oils, which are typically rich in long-chain PUFAs and low in saturated fats [55,56]. In contrast, although linseed and walnut oils have a low saturated fat content, they have a slightly higher saturated fat content than algal oils due to their botanical origin.

Monounsaturated fatty acids (MUFAs) were also significantly lower in the reformulated samples, especially in the algal oil group (10.60%) (*p* < 0.05). This is to be expected as linseed and algal oils naturally contain low levels of MUFAs compared to pork back fat [57]. The decrease in oleic acid (C18:1 n-9), the predominant MUFA in pork fat, was notable in all reformulated groups. Although C18:1 trans was detected in the reformulated samples (Table 2), it is worth noting that high-speed homogenisation can contribute to its formation through geometric isomerisation of unsaturated fatty acids [58], which should be taken into account when interpreting fatty acid profiles.

In contrast, the PUFA content increased significantly in the reformulated frankfurters (*p* < 0.05). The algal oil group had the highest PUFA content (80.58%), followed by linseed (64.25%) and walnut (61.61%). Linseed oil is particularly rich in alpha-linolenic acid (ALA, C18:3 n-3), which was the dominant n-3 fatty acid in the linseed oil group (47.53%), consistent with previous reports on linseed-enriched meat products [57,59]. Walnut oil also contributed to n-3 enrichment, but to a lesser extent, as it has higher levels of linoleic acid (C18:2 n-6) and moderate levels of ALA [60,61]. The algal oil group was notably enriched in long-chain n-3 fatty acids, particularly eicosapentaenoic acid (EPA, C20:5 n-3) and docosahexaenoic acid (DHA, C22:6 n-3), which are rarely found in vegetable oils but are of great benefit to cardiovascular health [26,55,56]. This finding demonstrates the biological advantage of microalgae oils over terrestrial vegetable oils in achieving marine-like n-3 PUFA profiles in meat products. However, it should be noted that the endogenous conversion of ALA to long-chain n-3 fatty acids such as EPA and DHA is limited in humans, with reported efficiencies typically below 10% [62], which may diminish the expected health benefits of linseed-enriched products.

The nutritional indices showed significant improvements in the reformulated frankfurters (Table 3). The Ʃn-6/Ʃn-3 ratio, an important nutritional indicator, improved significantly in the reformulated samples (*p* < 0.05). The control group had a ratio of 14.48, which is typical for pork-based products, while the flaxseed and algal oil groups achieved significantly lower ratios of 0.35 and 0.06, respectively. Ratios below 4:1 are considered beneficial for reducing the risk of chronic diseases, including cardiovascular disease [63]. Similar improvements in Ʃn-6/Ʃn-3 ratios have been found in meat products enriched with linseed and rapeseed oil [26,57].

The PUFA/SFA ratio exceeded the recommended ≥0.45 for a healthy diet in all reformulated groups [64], with the algal oil group achieving the highest ratio (>10), comparable to the results reported by Berasategi et al. [59] and Barros et al. [26] in algal oil-enriched emulsified meat.

The reformulated frankfurters exhibited significantly lower atherogenic (AI) and thrombogenic (TI) indices (*p* < 0.05), indicating a reduced potential for promoting atherosclerosis and thrombosis. These findings align with previous reports on meat products enriched with rapeseed and linseed oils [26,43,59]. Lower AI and TI values are considered beneficial, as they are linked to a decreased risk of cardiovascular disease [43,65]. Furthermore, the hypocholesterolaemic/hypercholesterolaemic (h/H) ratio, which reflects the proportion of fatty acids that support healthy cholesterol metabolism, showed a significant improvement across all reformulated groups, suggesting a more favourable lipid profile [26].

Cholesterol levels were significantly reduced in all reformulated samples (*p* < 0.05), with the lowest levels observed in the algal oil group, followed by the walnut and flaxseed groups (Table 3). This is consistent with previous studies in which the replacement of animal fats with vegetable oils reduced cholesterol levels by 45–50% [1,66]. Such a reduction is nutritionally relevant as excessive cholesterol intake is associated with increased cardiovascular risk [67].

Overall, these findings are consistent with previous research on vegetable oil and emulsion gel-based fat substitutes [1,12,23,50]. Importantly, the addition of algal oil not only increased PUFA content, but also provided unique long-chain n-3 fatty acids (EPA and DHA), which is a significant nutritional advantage over conventional vegetable oils [26,55,56].

### 3.3. Lipid Oxidation Assessment

**Figure 1:**
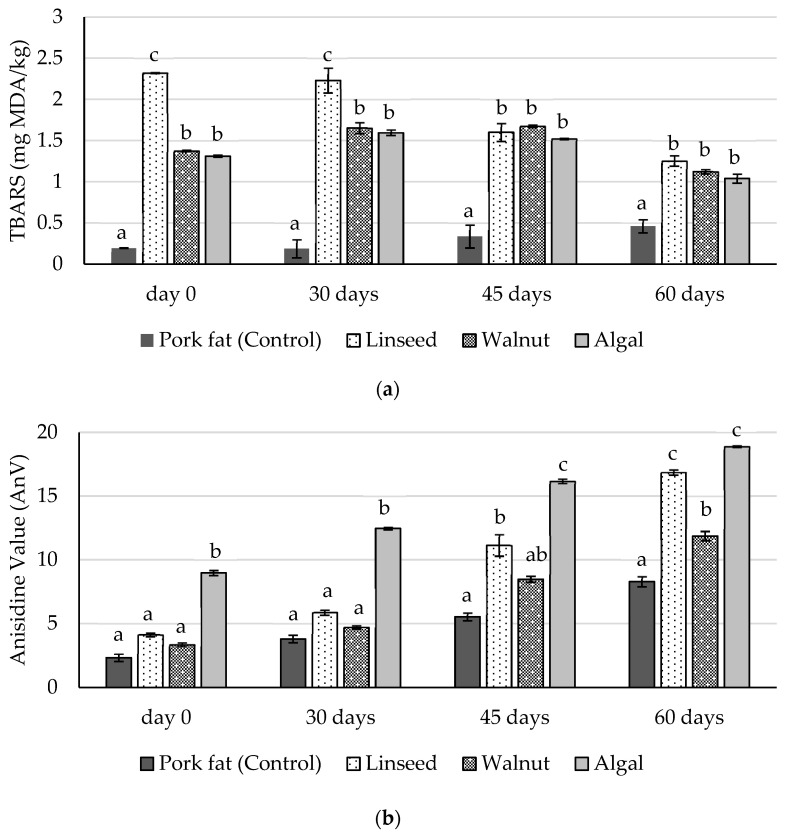
TBARS (**a**) and AnV (**b**) values of chicken frankfurters formulated with different fat sources during 60 days of cold storage under vacuum. Error bars represent standard deviation. Different letters (a–c) indicate significant differences (*p* < 0.05).

Lipid oxidation in the frankfurters was monitored by measuring TBARS and AnV over a period of 60 days of refrigerated vacuum storage (Figure 1). Throughout the storage period, the reformulated samples had significantly higher TBARS and AnV levels compared to the control (*p* < 0.05), indicating that the replacement of pork loin fat with polyunsaturated oil emulsions increases susceptibility to oxidative degradation. Notably, they were higher than the generally accepted limit of 1 mg MDA kg^−1^, indicating that the reformulated samples may have been more susceptible to sensory deterioration during storage. This result is consistent with previous studies showing that vegetable oil-enriched meat products are more susceptible to lipid oxidation due to their higher unsaturated fatty acid content [13,66,68].

Among the reformulated samples, the linseed oil group consistently had the highest TBARS values, especially on days 0 and 30, followed by the walnut oil and algal oil groups (*p* < 0.05), while the pork fat control had the lowest TBARS values across all time points (Figure 1a). Similar oxidative trends were reported by Choi et al. [1], where meat products made with vegetable oils had higher TBARS values than those made with animal fat. While the higher susceptibility of vegetable and marine oils to oxidation is primarily due to their higher PUFA content, the presence of pro-oxidising metal ions such as iron and copper can also significantly influence oxidation rates by catalysing peroxidation reactions, although these were not measured in the current study [69]. The elevated TBARS levels in the linseed oil samples can be directly linked to their extremely high content of α-linolenic acid (C18:3 n-3), which is highly susceptible to oxidative degradation [57]. These findings are consistent with those of Stajić et al. [70], who also reported increased oxidation in linseed oil-enriched frankfurters. Although walnut oil also contains significant amounts of polyunsaturated fatty acids (Table 2), the samples had intermediate TBARS levels—lower than the highly unstable linseed oil group, but higher than the control group (*p* < 0.05). Interestingly, on days 0 and 30, the algal oil samples had lower TBARS values than linseed group despite their high content of long-chain n-3 fatty acids such as eicosapentaenoic acid (EPA) and docosahexaenoic acid (DHA), which are themselves highly susceptible to oxidation [26]. This can be partly explained by possible differences in antioxidant content or the degree of oil refining, as certain algal oil formulations may contain antioxidants, such as tocopherols or carotenoids, that provide some oxidation protection [56,71].

In addition to TBARS, the anisidine value (AnV), which measures secondary oxidation products (mainly aldehydes), also increased during storage in all groups (Figure 1b), with the algal oil samples having the highest AnV values (*p* < 0.05). This is consistent with Fontes-Candia et al. [72], who showed that emulsified systems, especially those containing highly unsaturated oils, are susceptible to accelerated secondary oxidation. The high AnV value in the algal oil group also confirms the oxidative susceptibility of long-chain PUFAs such as EPA and DHA, which can generate significant amounts of aldehydes and other secondary oxidation products during degradation [26,69]. It is noteworthy that the walnut oil samples had the lowest AnV values among the reformulated groups (Figure 1b), indicating less formation of secondary oxidation products such as aldehydes. This suggests that walnut oil provides a better balance between oxidative stability and nutritional enhancement compared to the other reformulated samples.

It is important to note that the lack of added antioxidants in our formulations likely contributed to the accelerated lipid oxidation in all reformulated groups, which is consistent with the observations of Martínez et al. [73]. In addition, the emulsification process itself may have enhanced the oxidative reactions, as the formation of fine droplets increases the surface area of the oil and oxygen exposure, thus promoting oxidation [72]. While algal oils offer significant nutritional benefits, their high oxidative instability poses a technological challenge that requires careful formulation, possibly including the incorporation of antioxidants or encapsulation techniques to mitigate oxidation during storage [71,74]. Future studies should explore these protective strategies to improve the oxidative stability of frankfurters enriched with marine-derived oils.

### 3.4. Sensory Evaluation

**Figure 2:**
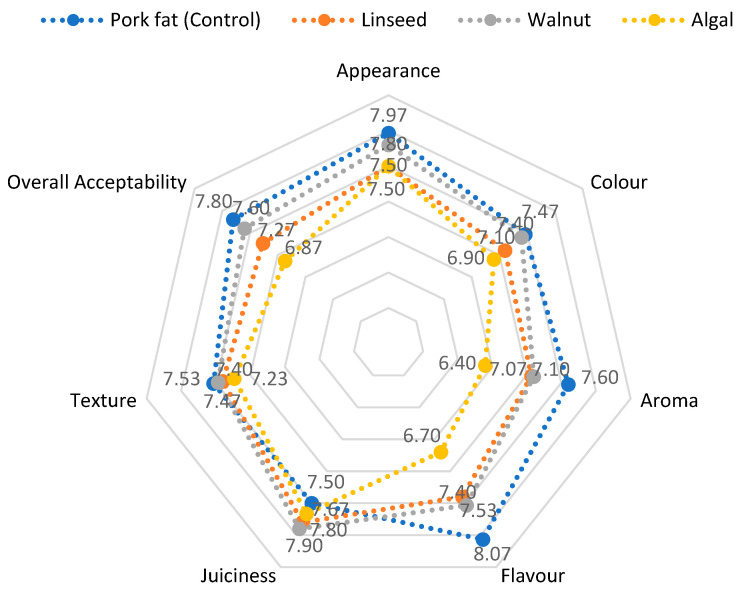
Sensory evaluation of chicken frankfurters formulated with different fat sources on a 9-point hedonic scale. The values on the diagram axes represent absolute mean values for the observed sensory attribute.

The sensory analysis using a 9-point scale showed that the control frankfurters consistently scored highest in the areas of appearance, flavour, taste and overall acceptability. Among the reformulated groups, the walnut oil samples scored the most favourable sensory ratings, while the algal oil samples scored the lowest, particularly for aroma and taste (Figure 2).

The lower appearance rating of the reformulated frankfurters is consistent with previous studies reporting that replacing pork fat with vegetable oils can negatively affect colour and visual appeal [1,50]. A similar deterioration in colour perception was found by Liu et al. [10] and Berasategi et al. [59] when using linseed or other vegetable oils in meat products.

In terms of flavour and juiciness, the walnut oil samples closely matched the control, which is consistent with previous findings that vegetable oil-based frankfurters can achieve sensory profiles comparable to high-fat products [1,50]. Linseed oil samples showed moderate acceptability but a lower taste rating, probably due to the high content of n-3 PUFA, which can cause a fishy odour and an oily aftertaste [75]. The algal oil samples had the lowest sensory scores, especially in terms of aroma and flavour, which is consistent with reports that marine oils can have off-flavours despite their nutritional value, likely due to the development of volatile oxidation products and the presence of fishy off-flavours commonly associated with marine-derived lipids [59]. Linseed oil also contributed to a mild fishy flavour, which is likely related to its high n-3 PUFA content, and affected taste acceptability. In contrast, the balanced fatty acid profile and milder flavour of walnut oil led to better sensory acceptability, suggesting that it is the most promising fat substitute among the oils tested. These results emphasise the importance of carefully selecting oil sources for fat replacement to balance health benefits with consumer sensory preferences.

Juiciness and texture remained relatively unaffected in all groups, with no significant sensory deterioration due to oil inclusion, confirming previous observations [59]. In the preference ratings, the walnut oil group was consistently favoured over the linseed oil and algal oil treatments, which is consistent with Choi et al. [1] and Méndez-Zamora et al. [51].

Although the reformulated samples generally scored lower than the control, all treatments remained within acceptable sensory limits, supporting the feasibility of using vegetable oils as a fat substitute in frankfurters. However, algal oil poses a sensory challenge as it has a strong impact on flavour and aroma (Figure 2).

### 3.5. Principal Component Analysis (PCA)

**Figure 3:**
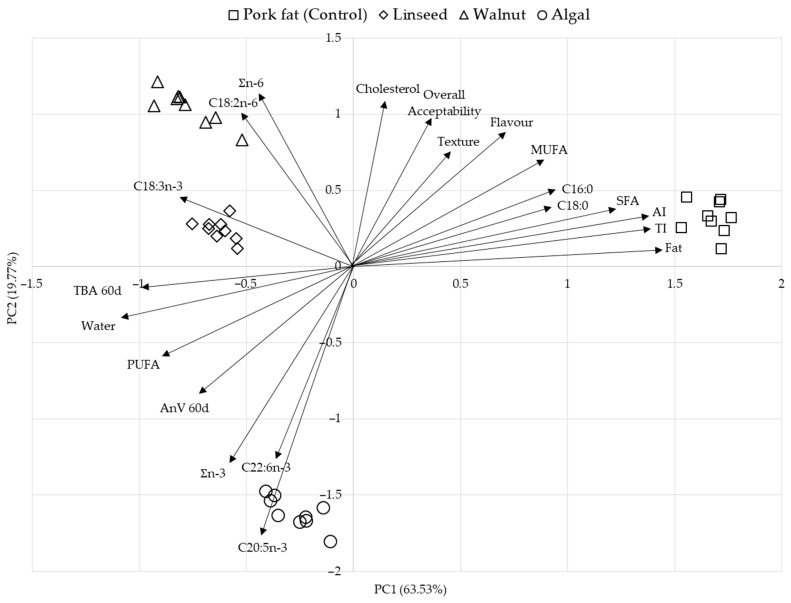
Principal component analysis (PCA) biplot of chicken frankfurters based on proximate composition, fatty acid profile, cholesterol content, lipid oxidation, and sensory attributes.

Principal component analysis (PCA) was performed to investigate the relationships between proximate composition, fatty acid profile, cholesterol content, lipid oxidation and sensory properties of chicken frankfurters produced with pork fat (control), linseed oil, walnut oil and algal oil emulsions (Figure 3). The first two main components together explained 83.30% of the total variance (PC1: 63.53%, PC2: 19.77%).

The control samples formed a distinct cluster strongly associated with higher total fat, saturated fat (C16:0, C18:0), cholesterol and increased atherogenic and thrombogenic indices, together with favourable sensory characteristics, such as texture, flavour, and overall acceptability. Conversely, samples of linseed and algal oil were more likely to be associated with healthier lipid characteristics, namely increased Ʃn-3 fatty acids and PUFA levels, although this was associated with less favourable sensory perceptions, particularly in the case of algal oil.

Interestingly, the walnut oil samples occupied an intermediate position, suggesting that they may offer the most favourable balance between nutritional improvement and sensory quality among the reformulated sausages (Figure 3). Their proximity to both sensory variables and improved fatty acid profiles suggests that walnut oil formulations could be a favoured alternative for the development of healthier sausages without significantly affecting consumer acceptance.

The PCA visualisation confirms the trade-off between improved health-related lipid indices and sensory appeal but also highlights walnut oil as a promising solution to this challenge.

## 4. Conclusions

This study shows that replacing pork fat with pre-emulsified vegetable and marine oils in chicken frankfurters significantly improves the nutritional profile by reducing total fat, saturated fat and cholesterol while enriching the product with health-promoting polyunsaturated fatty acids, especially n-3. Among the reformulated groups, walnut oil appeared to be the most balanced solution, offering nutritional improvements with minimal sensory compromise. Linseed oil offered stronger health benefits but slightly poorer sensory scores, while algal oil had the most favourable fatty acid profile but was least preferred in terms of taste and aroma. Importantly, the walnut oil group had the most favourable oxidation profile among the reformulated samples, with moderate levels of primary oxidation and the lowest accumulation of secondary oxidation products. This suggests that walnut oil may be the preferred choice for fat replacement as it effectively balances nutritional enhancement, oxidative stability and sensory quality. Although these differences were statistically significant, their nutritional relevance with regard to the long-term effects of diet should be investigated further. Principal component analysis supported these findings and showed that the walnut oil samples represented a promising middle ground between nutritional enhancement and sensory acceptability.

Future research should focus on optimising flavour in algal oil formulations and investigate the use of masking agents, antioxidants or flavour enhancers to improve sensory properties without compromising nutritional value. A limitation of this study is the absence of measurements of droplet size and polydispersity index for the emulsion gels, which are necessary to better understand their influence on product quality. In addition, consumer testing beyond trained panels, particularly with target market segments such as flexitarians, could provide deeper insights into product acceptance and purchase intent.

## Figures and Tables

**Table 1:** Proximate composition and energy value of chicken frankfurters formulated with different fat sources.

Parameters	Pork Fat (Control)	Linseed	Walnut	Algal
Water (%)	59.68 ± 2.45 ^a^	71.03 ± 3.27 ^b^	71.21 ± 1.47 ^b^	71.50 ± 4.52 ^b^
Protein (%)	15.15 ± 0.52 ^b^	14.48 ± 1.30 ^a^	14.46 ± 0.45 ^a^	14.48 ± 1.27 ^a^
Fat (%)	21.91 ± 2.60 ^b^	6.34 ± 1.06 ^a^	6.05 ± 0.73 ^a^	5.76 ± 0.59 ^a^
Ash (%)	2.98 ± 0.23	2.82 ± 0.26	2.72 ± 0.27	2.82 ± 0.33
Carbohydrates (%)	0.29 ± 0.06 ^a^	5.34 ± 0.69 ^b^	5.55 ± 1.03 ^b^	5.44 ± 0.76 ^b^
Energy value (kcal/100 g)	257.74 ± 4.45 ^c^	128.29 ± 1.70 ^b^	126.18 ± 2.17 ^ab^	123.41 ± 4.30 ^a^

^a–c^ Different letters within the same row denote significant differences between means at *p* < 0.05.

**Table 2:** Fatty acid composition (g/100 g total fatty acids) of chicken frankfurters formulated with different fat sources.

Fatty Acid	Pork Fat (Control)	Linseed	Walnut	Algal
C12:0	0.17 ± 0.02 ^b^	0.11 ± 0.01 ^a^	0.24 ± 0.02 ^c^	0.36 ± 0.01 ^d^
C14:0	1.41 ± 0.07 ^c^	0.17 ± 0.01 ^a^	0.23 ± 0.02 ^a^	0.49 ± 0.02 ^b^
C16:0	25.58 ± 1.66 ^c^	8.13 ± 0.53 ^b^	9.24 ± 0.24 ^b^	4.60 ± 0.43 ^a^
C16:1	2.03 ± 0.24 ^c^	0.52 ± 0.01 ^a^	0.71 ± 0.07 ^b^	0.64 ± 0.05 ^ab^
C17:0	0.60 ± 0.03 ^c^	0.07 ± 0.01 ^a^	0.11 ± 0.04 ^ab^	0.11 ± 0.01 ^b^
C17:1	0.54 ± 0.04 ^c^	0.01 ± 0.04 ^a^	0.10 ± 0.01 ^b^	0.01 ± 0.02 ^a^
C18:0	14.04 ± 1.83 ^d^	5.03 ± 0.09 ^c^	3.36 ± 0.19 ^b^	1.81 ± 0.09 ^a^
C18:1 trans	0.00 ± 0.00 ^a^	0.14 ± 0.05 ^c^	0.12 ± 0.05 ^c^	0.05 ± 0.01 ^b^
C18:1 n-9	37.25 ± 2.99 ^c^	21.14 ± 2.70 ^b^	23.01 ± 2.24 ^b^	7.61 ± 0.86 ^a^
C18:2 n-6	14.73 ± 2.15 ^b^	16.21 ± 1.87 ^b^	51.78 ± 3.49 ^c^	4.04 ± 0.50 ^a^
C20:0	0.05 ± 0.00	0.07 ± 0.12	0.00 ± 0.01	0.03 ± 0.00
C18:3 n-6	0.15 ± 0.02 ^b^	0.22 ± 0.07 ^c^	0.06 ± 0.01 ^a^	0.20 ± 0.02 ^bc^
C18:3 n-3	0.92 ± 0.05 ^a^	47.53 ± 2.18 ^c^	9.48 ± 0.66 ^b^	0.30 ± 0.03 ^a^
C20:1	0.58 ± 0.05 ^c^	0.00 ± 0.00 ^a^	0.02 ± 0.01 ^a^	0.10 ± 0.01 ^b^
C20:2 n-6	0.53 ± 0.04 ^b^	0.09 ± 0.01 ^a^	0.11 ± 0.01 ^a^	0.08 ± 0.01 ^a^
C22:0	0.04 ± 0.01 ^b^	0.06 ± 0.02 ^b^	0.01 ± 0.01 ^a^	0.05 ± 0.01 ^b^
C20:3 n-6	0.12 ± 0.02 ^b^	0.09 ± 0.02 ^a^	0.08 ± 0.01 ^a^	0.21 ± 0.02 ^c^
C20:3 n-3	0.11 ± 0.02 ^b^	0.04 ± 0.01 ^ab^	0.01 ± 0.00 ^a^	0.06 ± 0.05 ^ab^
C22:1 n-9	0.46 ± 0.02 ^a^	0.55 ± 0.02 ^b^	0.49 ± 0.02 ^ab^	2.21 ± 0.10 ^c^
C20:5 n-3	0.02 ± 0.00 ^a^	0.03 ± 0.00 ^a^	0.06 ± 0.01 ^a^	10.98 ± 1.61 ^b^
C22:6 n-3	0.03 ± 0.00 ^a^	0.04 ± 0.00 ^a^	0.03 ± 0.00 ^a^	64.73 ± 3.42 ^b^

^a–d^ Different letters within the same row denote significant differences between means at *p* < 0.05.

**Table 3:** Nutritional indices and cholesterol content of chicken frankfurters formulated with different fat sources.

Parameters *	Pork Fat (Control)	Linseed	Walnut	Algal
SFA (%)	42.02 ± 3.71 ^c^	13.67 ± 1.56 ^b^	13.24 ± 1.36 ^b^	7.76 ± 1.43 ^a^
MUFA (%)	40.88 ± 2.24 ^c^	22.23 ± 2.69 ^b^	24.35 ± 1.24 ^b^	10.60 ± 1.44 ^a^
PUFA (%)	16.60 ± 2.17 ^a^	64.25 ± 3.66 ^b^	61.61 ± 4.03 ^b^	80.58 ± 5.54 ^c^
PUFA/SFA	0.40 ± 0.07 ^a^	4.62 ± 0.35 ^b^	4.67 ± 0.53 ^b^	10.50 ± 1.19 ^c^
Ʃn-6 (%)	15.53 ± 3.17 ^b^	16.61 ± 2.87 ^b^	52.03 ± 4.48 ^c^	4.52 ± 0.91 ^a^
Ʃn-3 (%)	1.07 ± 0.04 ^a^	47.63 ± 3.19 ^c^	9.58 ± 1.66 ^b^	76.06 ± 4.46 ^d^
Ʃn-6/Ʃn-3	14.48 ± 2.21 ^c^	0.35 ± 0.02 ^a^	5.44 ± 0.43 ^b^	0.06 ± 0.01 ^a^
AI	0.55 ± 0.05 ^c^	0.10 ± 0.01 ^ab^	0.12 ± 0.02 ^b^	0.08 ± 0.01 ^a^
TI	1.31 ± 0.13 ^c^	0.08 ± 0.01 ^a^	0.19 ± 0.02 ^b^	0.03 ± 0.00 ^a^
h/H	2.00 ± 0.17 ^a^	10.32 ± 1.63 ^b^	8.95 ± 1.47 ^b^	17.43 ± 1.47 ^c^
Cholesterol (mg/100 g)	61.55 ± 3.00 ^c^	54.43 ± 3.96 ^b^	56.82 ± 2.14 ^b^	42.55 ± 3.12 ^a^

* SFA—saturated fatty acids; MUFA—monounsaturated fatty acids; PUFA—polyunsaturated fatty acids; Ʃn-6—sum of all n-6 fatty acids; Ʃn-3—sum of all n-3 fatty acids; AI—Atherogenic Index; TI—Thrombogenic Index; h/H—hypocholesterolemic/hypercholesterolemic ratio. ^a–d^ Different letters within the same row denote significant differences between means at *p* < 0.05.

## Data Availability

The original contributions presented in this study are included in the article. Further inquiries can be directed to the corresponding author.
